# Kelch-like ECT2-interacting protein KLEIP regulates late-stage pulmonary maturation via Hif-2α in mice

**DOI:** 10.1242/dmm.014266

**Published:** 2014-05-01

**Authors:** Nicole Woik, Christian T. Dietz, Kathrin Schäker, Jens Kroll

**Affiliations:** 1 Department of Vascular Biology and Tumor Angiogenesis, Center for Biomedicine and Medical Technology Mannheim (CBTM), Medical Faculty Mannheim of Heidelberg University, 68167 Mannheim, Germany.; 2 Division of Vascular Oncology and Metastasis, German Cancer Research Center (DKFZ-ZMBH Alliance), 69120 Heidelberg, Germany.

**Keywords:** BTB-kelch protein KLEIP, Respiratory distress syndrome, Endothelial cells, Betamethasone, Hif-2α

## Abstract

Respiratory distress syndrome (RDS) caused by preterm delivery is a major clinical problem with limited mechanistic insight. Late-stage embryonic lung development is driven by hypoxia and the hypoxia-inducible transcription factors Hif-1α and Hif-2α, which act as important regulators for lung development. Expression of the BTB-and kelch-domain-containing (BTB-kelch) protein KLEIP (Kelch-like ECT2-interacting protein; also named Klhl20) is controlled by two hypoxia response elements, and KLEIP regulates stabilization and transcriptional activation of Hif-2α. Based on the available data, we hypothesized an essential role for KLEIP in murine lung development and function. Therefore, we have performed a functional, histological, mechanistic and interventional study in embryonic and neonatal *KLEIP*^−/−^ mice. Here, we show that about half of the *KLEIP^−/−^* neonates die due to respiratory failure that is caused by insufficient aeration, reduced septal thinning, reduced glycogenolysis, type II pneumocyte immaturity and reduced surfactant production. Expression analyses in embryonic day (E) 18.5 lungs identified KLEIP in lung capillaries, and showed strongly reduced mRNA and protein levels for Hif-2α and VEGF; such reduced levels are associated with embryonic endothelial cell apoptosis and lung bleedings. Betamethasone injection in pregnant females prevented respiratory failure in *KLEIP^−/−^* neonates, normalized lung maturation, vascularization, aeration and function, and increased neonatal Hif-2α expression. Thus, the experimental study shows that respiratory failure in *KLEIP^−/−^* neonates is determined by insufficient angiocrine Hif-2α–VEGF signaling and that betamethasone activates this newly identified signaling cascade in late-stage embryonic lung development.

## INTRODUCTION

Preterm births represent 12–13% in the USA and 5–9% in many other developed countries (Goldenberg et al., 2008). Respiratory distress syndrome (RDS) is the most common cause of respiratory distress in newborns that is triggered by lung prematurity, and has high mortality rates (Ghafoor et al., 2003). Therapeutic treatment of RDS includes glucocorticoids, surfactants and mechanical ventilation, which can prevent newborns from dying. Glucocorticoids are able to reduce the risk for neonatal RDS and death, and can accelerate lung maturation (Crowther et al., 2006; Roberts and Dalziel, 2006). However, only very little is known so far about the direct effects and long term risks of this treatment. Nowadays, two glucocorticoids are approved for standard antenatal therapy: dexamethasone and betamethasone. Treatment conditions are still controversially discussed, because some experimental studies indicated adverse effects, including fetal growth restriction (French et al., 1999; Ikegami et al., 1997; Jobe et al., 1998), hypertension (Derks et al., 1997) and neurodevelopmental defects such as reduced brain growth or myelination (Dunlop et al., 1997; Huang et al., 1999). Even though glucocorticoid treatment has improved antenatal therapy, the actual challenge is to understand both the molecular mechanisms of late-stage lung development and the molecular pathways of glucocorticoid action to define new targeted strategies to avoid neonatal RDS and death.

Breathing at birth requires prenatal surfactant production and septal thinning. Surfactant reduces alveolar surface tension at the air-liquid interface of the lungs and prevents alveolar collapse (Whitsett et al., 2010). Surfactant consists of phospholipids and the surfactant proteins (SPs) SP-A, SP-B, SP-C and SP-D (Rooney et al., 1994). Surfactant proteins are mainly produced by type II pneumocytes, which also accumulate glycogen deposits for the synthesis of phospholipids of pulmonary surfactant (Bourbon et al., 1982). Type II pneumocytes differentiate during the last phase of embryonic lung development, called the saccular stage, and give rise to type I pneumocytes, which line the alveoli and mediate gas exchange (Huang et al., 2012). At this time point, alveoli are separated by thick septa. Septal thinning occurs shortly before birth through alveolar dilatation and capillary remodeling (ad hoc Statement Committee, 2004).

Late-stage lung development is driven by hypoxia (Land and Wilson, 2005), and hypoxia-inducible transcription factors Hif-1α and Hif-2α (also known as endothelial-PAS-domain protein 1) act as key regulators in epithelial, mesenchymal and vascular morphogenesis (Saini et al., 2008; Wagner et al., 2004). Both are in their active state heterodimerized to ARNT-1 (aryl hydrocarbon receptor nuclear translocator or Hif-1β) (Shimoda and Semenza, 2011; Tian et al., 1997). Whereas Hif-1α expression decreases quickly after birth, Hif-2α upregulation starts in lung at the saccular stage and remains until adulthood (Rajatapiti et al., 2008). Absence of Hif-2α in mice leads to partial embryonic lethality due to vascular defects (Peng et al., 2000) or bradycardia (Tian et al., 1998), and surviving embryos suffer from neonatal death due to impaired lung maturation (Compernolle et al., 2002) or from multiorgan failure (Scortegagna et al., 2003). The importance of Hif-2α for late-stage type II pneumocyte development was monitored in different loss-of-function models (Compernolle et al., 2002; Huang et al., 2012; Skuli et al., 2009; Wagner et al., 2004). Hif-2α controls type II pneumocyte maturation and, consequently, surfactant production and type I pneumocyte differentiation (Huang et al., 2012). In addition, Hif-2α is a transcriptional activator for VEGF, which thereby drives prenatal pulmonary vascularization and capillary remodeling. Moreover, VEGF determines epithelial maturation (Akeson et al., 2005; Akeson et al., 2003; Compernolle et al., 2002; Del Moral et al., 2006; Zhao et al., 2005) and administration of VEGF is found to rescue Hif-2α deficiency (Compernolle et al., 2002). In the development of other organs, VEGF acts as an endothelial-derived angiocrine factor, and regulates tissue differentiation and maturation (Butler et al., 2010b; Lammert et al., 2003). In lung regeneration, angiocrine signaling is linked to MMP14 (Ding et al., 2011).

**TRANSLATIONAL IMPACT**

**Clinical issue**

Preterm birth, which accounts for 12–13% of births in the USA, can cause early neonatal death. As the leading cause of fatality after preterm delivery, respiratory distress syndrome (RDS) is a major clinical problem. The syndrome is thought to be caused by insufficient developmental production of lung surfactant, in addition to structural immaturity of neonatal lungs. Women who are at risk of preterm delivery are usually treated with glucocorticoids, a group of hormones that enhance the production of surfactant. However, prenatal glucocorticoid treatment could be associated with adverse effects and long-term complications, so there is a need to understand the basic mechanisms of late-stage lung maturation and develop new targeted therapies. Hypoxia is involved in lung development, and KLEIP, a hypoxia-inducible protein that belongs to the functionally diverse family of kelch proteins, is thought to play an important, hitherto undefined, role in prenatal development.

**Results**

Here, the authors use mice to determine whether KLEIP could play a role in lung maturation. The authors report that several *KLEIP^−/−^* neonates incur early neonatal death because of respiratory failure. Respiratory failure in *KLEIP^−/−^* neonates is caused by lung immaturity, marked by non-functional pneumocytes (alveolar cells) and loss of surfactant production. KLEIP is expressed in lung capillaries, where its absence leads to endothelial cell apoptosis and lung bleedings. Expression analyses for Hif-2α (the interaction partner of KLEIP) and its target VEGF showed reduced embryonic expression of Hif-2α and VEGF in *KLEIP^−/−^* lungs. Reduced embryonic VEGF expression correlated with attenuated pneumocyte differentiation and respiratory failure. Respiratory failure in *KLEIP^−/−^* neonates could be rescued by embryonic glucocorticoid application, which, remarkably, was shown to increase Hif-2α expression. This suggests that pneumocyte immaturity in *KLEIP^−/−^* mice is a result of non-functional angiocrine signaling via Hif-2α and VEGF.

**Implications and future directions**

This study provides evidence of a role for KLEIP in late-stage embryonic lung maturation, and suggests that its interaction with Hif-2α triggers a signaling cascade that is indispensable for normal lung development and maturation. The data further show that expression levels of KLEIP and Hif-2α in embryonic lungs determine whether respiratory failure develops after birth. Lastly, the glucocorticoid betamethasone, which is commonly used in clinics to prevent neonatal RDS, was shown to act on the newly identified signaling cascade involved in embryonic lung development. Thus, the study provides insights into the mechanism of action of glucocorticoid treatment and describes a new animal model for the investigation of potential targeted therapies for RDS.

Expression of the BTB- and kelch-domain-containing (BTB-kelch) protein KLEIP (Kelch-like ECT2-interacting protein; also named Klhl20) is controlled by two hypoxia response elements that bind Hif-1α (Yuan et al., 2011). Therefore, KLEIP expression is strongly hypoxia-dependent (Nacak et al., 2007; Yuan et al., 2011). *In vitro* data further suggest a function for KLEIP in stabilization and transcriptional activation of Hif-2α protein (Higashimura et al., 2011). In addition, KLEIP has been shown to regulate human vascular endothelial cell function through RhoA activation (Nacak et al., 2007). Gene targeting studies in adult *KLEIP^−/−^* mice have recently shown that KLEIP regulates corneal epithelial integrity (Hahn et al., 2012). However, because about 50% of *KLEIP^−/−^* mice die within the first few days after birth, this study suggests an additional, earlier function for KLEIP during embryonic or postnatal development. In order to identify KLEIP’s function in early mouse development, we have analyzed embryonic and neonatal *KLEIP^−/−^* mice. We show that several *KLEIP^−/−^* neonates die directly after birth due to severe respiratory failure that is linked to impaired vascular remodeling and reduced surfactant production owing to altered Hif-2α and VEGF expression. Injection of betamethasone in pregnant mice rescued respiratory failure in *KLEIP^−/−^* neonates, thereby describing the KLEIP–Hif-2α axis as a newly identified target for betamethasone.

## RESULTS

### Respiratory distress, cyanosis and neonatal death in *KLEIP^−/−^* mice

**Fig. 1.**
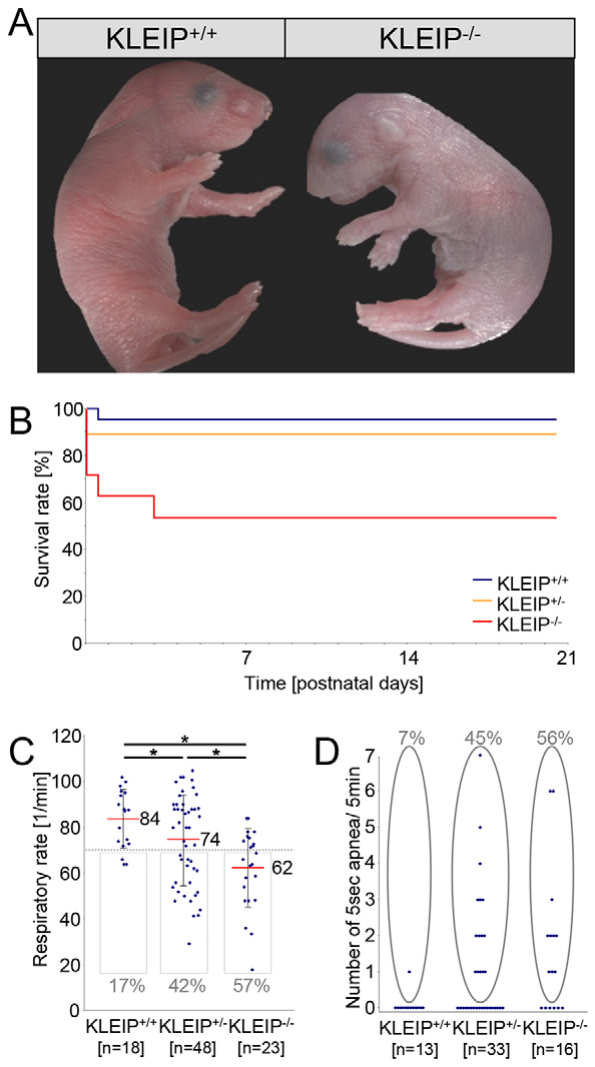
**Mortality is associated with respiratory failure in *KLEIP^−/−^ mice*.** (A) Respiratory failure results in cyanotic skin oxygenation in *KLEIP^−/−^* newborns. *KLEIP^+/+^* littermates served as controls. (B) Survival curve for *KLEIP^+/+^*, *KLEIP^+/−^* and *KLEIP^−/−^* mice shows increased neonatal lethality for *KLEIP^−/−^* mice. 38% of *KLEIP^−/−^* neonates and 12% of *KLEIP^+/−^* neonates died within the first 12 hours after birth (*n*=25 per group). (C) Reduced medium respiratory rate in *KLEIP^−/−^* (62 minute^−1^) and *KLEIP^+/−^* neonates (74 minute^−1^) as compared with their wild-type littermates (84 minute^−1^). 57% of *KLEIP^−/−^* and 42% of *KLEIP^+/−^* neonates show respiratory rates beneath the physiological respiratory limit of 70 minute^−1^ (gray border) (Ewringmann, 2008). (D) Regular breathing of *KLEIP^−/−^* neonates is interrupted by periods of apnea. Apnea is recorded for 56% of *KLEIP^−/−^* and 45% of *KLEIP^+/−^* but only for 7% of *KLEIP^+/+^* neonates. Whereas apnea in *KLEIP^+/+^* mice is restricted to one breathing arrest within 5 minutes, *KLEIP^+/−^* and *KLEIP^−/−^* newborns show irregular breathing activity including several arrests. *P<0.05.

We have previously generated *B6.129-KLEIP^tm/Mhm^* mice and we have shown that adult *KLEIP^−/−^* mice progressively develop a corneal opacity (Hahn et al., 2012). During this previous study we also noticed that ~50% of *KLEIP^−/−^* mice die within the first few days after birth, yet reasons for this reduced viability remained unclear. In order to clarify the function of KLEIP during embryonic and postnatal development, we have performed a detailed analysis of *KLEIP^−/−^* embryos and newborn mice. Before embryonic day (E) 18.5 of embryonic development we observed a slightly reduced number of *KLEIP^−/−^* embryos (supplementary material Fig. S1A) and histological analyses at different embryonic stages revealed bleedings in few *KLEIP^−/−^* embryos, which probably cause early embryonic death (supplementary material Fig. S2). Strikingly, on postnatal day (P)0, the number of *KLEIP^−/−^* pups originating from *KLEIP^+/−^* breeding diminished suddenly to 15% (supplementary material Fig. S1A), which suggested an early neonatal death. Indeed, several *KLEIP^−/−^* newborns remained cyanotic after birth and gasped for air, whereas their corresponding wild-type littermates (*KLEIP^+/+^*) acquired the characteristic pink color after a few breaths of air and moved actively (Fig. 1A). As a consequence, 38% of *KLEIP^−/−^* pups died within the first 12 hours of life, of which 26% died within the first 6 hours (Fig. 1B). This observation suggested a respiratory failure in *KLEIP^−/−^* neonates. In order to characterize respiratory failure in *KLEIP^−/−^* mice, breathing activity was determined in *KLEIP^+/+^* and *KLEIP^−/−^* newborns. *KLEIP^−/−^* newborns significantly suffered from a decreased respiratory rate of 62 breaths per minute compared with 74 and 84 breaths per minute in *KLEIP^+/−^* and *KLEIP^+/+^* neonates, respectively (Fig. 1C). Notably, the decreased respiratory rate in *KLEIP^−/−^* mice is below the physiological respiratory limit of 70 minute^−1^ and goes along with poor survival (Ewringmann, 2008). Furthermore, breathing in neonatal *KLEIP^−/−^* mice was interrupted by several episodes of apnea, which was classified as a breathing arrest of more than 5 seconds. For 56% of all *KLEIP^−/−^* mice and 45% of all *KLEIP^+/−^* mice, breathing arrests were recorded. For *KLEIP^+/+^* neonates, only 7% breathing arrests were observed and apnea was restricted to only one time within 5 minutes, whereas KLEIP^+/−^ and *KLEIP^−/−^* pups showed up to seven arrests within the same period of time (Fig. 1D). The respiratory analyses were completed by findings that lungs of *KLEIP^−/−^* neonates suffering from severe respiratory failure were insufficiently aerated and did not float in PBS solution as had been observed for *KLEIP^+/+^* lungs (supplementary material Fig. S1B).

### Respiratory musculature and closure of ductus arteriosus are unaffected in *KLEIP^−/−^* mice

**Fig. 2.**
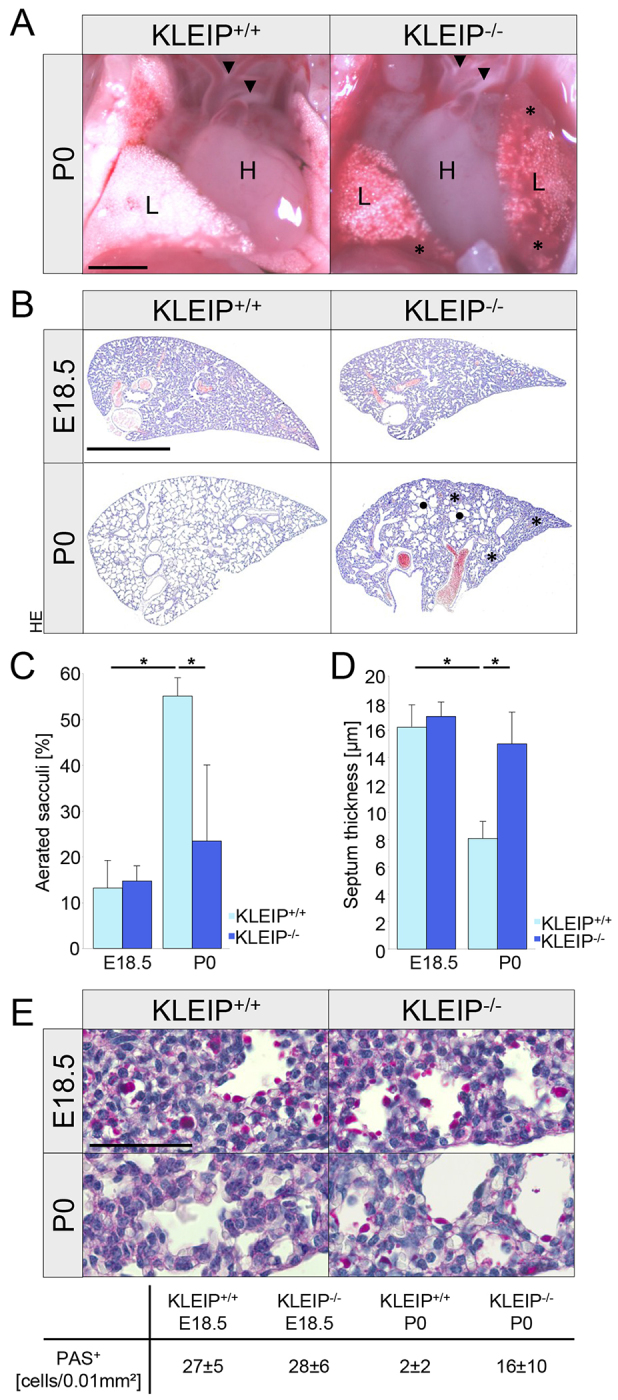
**Decreased ventilation and reduced glycogenolysis in *KLEIP^−/−^* neonates.** (A) Comparative analysis of P0 *KLEIP^+/+^* and *KLEIP^−/−^* lungs shows large unventilated areas in *KLEIP^−/−^* neonates (asterisks). After birth, the ductus arteriosus in *KLEIP^+/+^* and *KLEIP^−/−^* pups is closed and aortic arch architecture is developed normally (arrowheads). L: lung, H: heart. Scale bar: 1 mm. (B) Histological analysis of *KLEIP^−/−^* and *KLEIP^+/+^* lungs at prenatal (E18.5) and neonatal (P0) stages demonstrates lung maturation during the saccular stage. Whereas *KLEIP^+/+^* and *KLEIP^−/−^* lungs show no histological differences at E18.5, P0 *KLEIP^−/−^* lungs display large unaerated (asterisks) and inflated (points) areas. HE: haemalaun-eosin. Scale bar: 1 mm. (C) Quantification of unfolded sacculi (*n*≥4). After birth, quantification indicates reduced aeration of *KLEIP^−/−^* lungs (22%) in comparison to *KLEIP^+/+^* lungs (57%). Whereas *KLEIP^+/+^* lungs showed a significant increase of aerated sacculi from E18.5 to P0, *KLEIP^−/−^* lungs did not. (D) At E18.5, septum thickness is the same in *KLEIP^+/+^* and *KLEIP^−/−^* lungs (*n*≥4). At P0, septum thickness decreases to 8 μm in *KLEIP^+/+^* neonates, whereas, in *KLEIP^−/−^* newborns, septae still show a median thickness of 15 μm. (E) PAS staining shows equal glycogen deposits in *KLEIP^+/+^* and *KLEIP^−/−^* lungs at E18.5. Although, in *KLEIP^+/+^* lungs, glycogen disappears at P0 (2±2 PAS^+^ cells/0.01 mm^2^), *KLEIP^−/−^* neonates still show abundant glycogen granules (16±10 PAS^+^ cells/0.01 mm^2^). *n*=5; scale bar: 50 μm. *P<0.05.

Respiratory musculature is essential for physiological respiration. To exclude breathing failure in *KLEIP^−/−^* mice due to diaphragmic hernia, *KLEIP^−/−^* mice were examined. However, anatomical differences were not observed as compared with *KLEIP^+/+^* littermates. Furthermore, nasal obstruction and developmental retardation as a possible reason for respiratory failure could be excluded (supplementary material Fig. S3A–C). Lastly, we addressed the question of whether cardiovascular abnormalities could be the primary cause of respiratory distress and cyanosis in *KLEIP^−/−^* mice as has been described for Foxc2- and Sema3c-deficient single-mutant mice (Feiner et al., 2001; Iida et al., 1997). However, we did not observe evidence for aortic arch abnormalities or persistent ductus arteriosus (Fig. 2A). Thus, respiratory failure in *KLEIP^−/−^* mice is not caused by growth retardation, abnormalities of large blood vessels, diaphragmic hernia or airway closure.

### KLEIP deficiency in mice results in insufficient aeration, increased septal thickness and reduced surfactant production

**Fig. 3.**
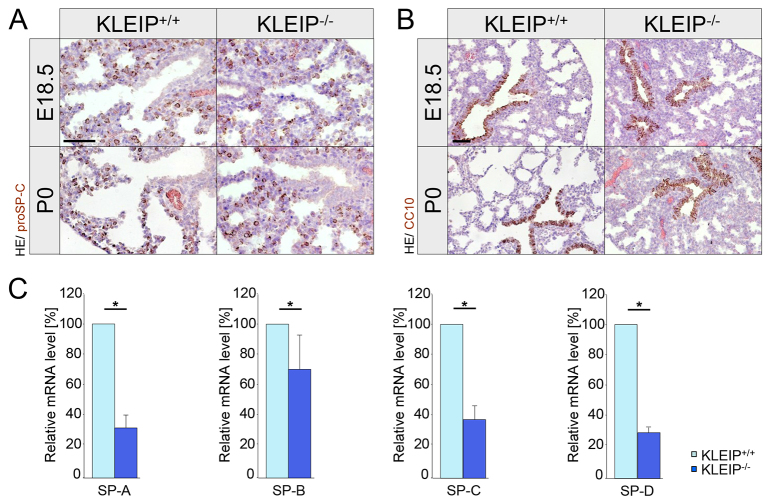
**Respiratory distress in *KLEIP^−/−^* mice is associated with reduced surfactant expression.** (A) Staining for type II pneumocytes (pro SP-C) and (B) Clara cells (CC10) shows normal formation of these cell types in *KLEIP^+/+^* and *KLEIP^−/−^* E18.5 embryos and P0 neonates. Presence of type II pneumocytes but reduced surfactant formation (shown in C) indicates immature and non-functional type II pneumocytes. HE: haemalaun-eosin. (C) Expression analysis for surfactant proteins at E18.5 shows reduced mRNA expression for *SP-A*, *SP-B*, *SP-C* and *SP-D* in embryonic *KLEIP^−/−^* lungs, indicating type II pneumocyte immaturity. *n*=7; controls in A and B were negative for HRP. Scale bars: 50 μm. *P<0.05.

At birth, we observed large unventilated areas in *KLEIP^−/−^* mice, indicating insufficient embryonic lung maturation (Fig. 2A). Histological characterization of lung development revealed no major differences in *KLEIP^−/−^* and *KLEIP^+/+^* mice at pseudoglandular stage (E13.5), canalicular stages (E16.5-E17.5) and early saccular stage (E18.5), pointing to a late-stage maturation deficiency in *KLEIP^−/−^* mice (Fig. 2B). Strikingly, at birth more than 30% of *KLEIP^−/−^* lungs showed large unaerated areas in comparison to the uniformly ventilated *KLEIP^+/+^* lungs (Fig. 2B). Notably, some neonatal *KLEIP^−/−^* lungs strongly resembled the lung appearance at E18.5 and did not show any signs of aeration. Quantification for lung development after birth resulted in 54% lung aeration in *KLEIP^+/+^* lungs. In contrast, newborn *KLEIP^−/−^* mice achieved only median dilatation of 22%, which does not represent a significant change to E18.5 (Fig. 2C). At the saccular stage, both *KLEIP^+/+^* and *KLEIP^−/−^* embryos showed comparable terminal sacs and alveolar septa (Fig. 2B–D). However, at birth, thinning of alveolar septa and mesenchyme, which is essential for neonatal blood-gas exchange, did not occur in *KLEIP^−/−^* neonates (Fig. 2B,D). These data indicate that KLEIP directly affects lung aeration and breathing but does not regulate early embryonic lung development. Therefore, pneumocyte differentiation and vascular remodeling as key processes within late-stage lung maturation were further analyzed. Pneumocyte-derived glycogen granula are important prerequisites for lung maturation and serve as a source for late-stage surfactant production (Bourbon et al., 1982). At the saccular stage, both *KLEIP^+/+^* and *KLEIP^−/−^* lungs were positive for Periodic acid-Schiff (PAS) (27±5 versus 28±6 PAS-positive cells); however, after birth, PAS-positive cells only disappeared in *KLEIP^+/+^* lungs but were still detectable in *KLEIP^−/−^* lungs (*KLEIP^+/+^* 2±2 versus *KLEIP^−/−^* 16±10). This specified immaturity and dysfunction of type II pneumocytes in *KLEIP^−/−^* mice, which were not able to convert glycogen deposits into surfactant (Fig. 2E). Interestingly, immunohistochemistry revealed no reduction in the number of type II pneumocytes (Fig. 3A; supplementary material Fig. S3D, left). In addition, Clara cells appeared normal in *KLEIP^+/+^* and *KLEIP^−/−^* mice (Fig. 3B; supplementary material Fig. 3D, right). To further analyze immaturity and dysfunction of type II pneumocytes in *KLEIP^−/−^* lungs, mRNA expression analysis for surfactant proteins SP-A, SP-B, SP-C and SP-D was performed (Fig. 3C). Strikingly, *SP-A*, *SP-B*, *SP-C* and *SP-D* expressions were prenatally strongly downregulated, endorsing the observation that type II pneumocytes in *KLEIP^−/−^* lungs are non-functional. Taken together, the data suggest a function for KLEIP in type II pneumocyte maturation and surfactant production.

### Respiratory failure in *KLEIP^−/−^* mice is linked to regressive vascularization and reduced Hif-2α and VEGF expression that affects capillary remodeling and lung maturation

**Fig. 4.**
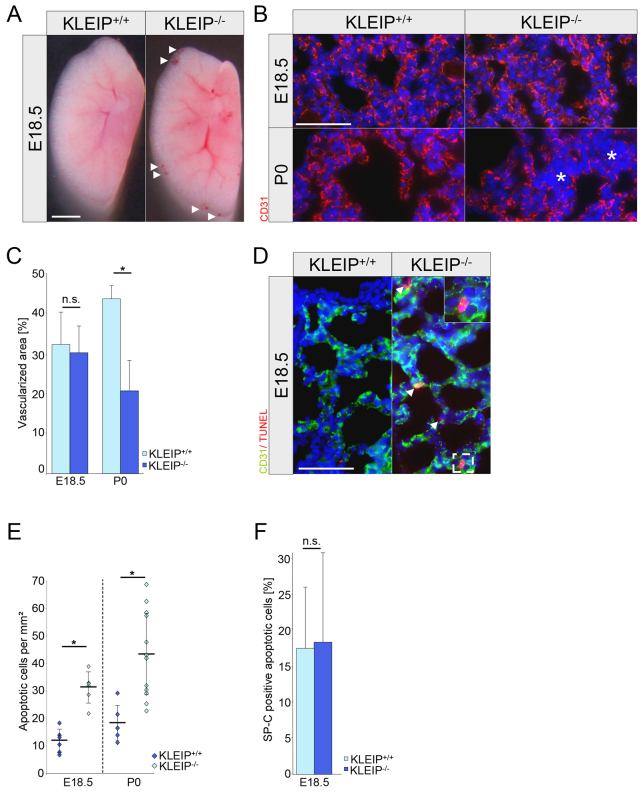
***KLEIP^−/−^* lungs show enhanced apoptosis and regressive alveolar vascularization.** (A) Lung morphology at E18.5 shows minor capillary lung bleedings (arrowheads) in *KLEIP^−/−^* embryos. Scale bar: 1 mm. (B,C) Lung capillarization (CD31 staining) in *KLEIP^−/−^* and *KLEIP^+/+^* lungs is comparable at E18.5. At P0, *KLEIP^−/−^* lungs show vascular regression marked by avascular areas (asterisks). *n*≥5. Scale bar: 50 μm. (D) Double immunostaining for apoptosis (TUNEL) and endothelial cells (CD31) to detect vascular apoptosis. Apoptosis is predominantly found in lung capillaries of *KLEIP^−/−^* neonates (arrowheads). Lung capillaries of *KLEIP^+/+^* neonates are negative for endothelial apoptosis. Scale bar: 50 μm. Boxed area is enlarged in the top right. (E) Quantification of TUNEL staining reveals a threefold increased apoptotic rate in E18.5 *KLEIP^−/−^* lungs and a twofold increased apoptotic rate in P0 *KLEIP^−/−^* lungs; *n*≥6. (F) Quantification of TUNEL/SP-C double-positive cells at E18.5 shows no increased apoptosis in type II pneumocytes (*n*=5 animals per group). n.s.: not significant. *P<0.05.

Embryonic vascular development during lung formation was indistinguishable in *KLEIP^+/+^* and *KLEIP^−/−^* embryos. At E18.5, few *KLEIP^−/−^* mice showed weak spontaneous bleedings that were restricted to distal parts of the lobes (Fig. 4A); however, endothelial CD31 (cluster of differentiation 31) staining did not detect differences in lung vascularization in *KLEIP^+/+^* and *KLEIP^−/−^* lungs at this embryonic stage (Fig. 4B,C). In contrast, P0 *KLEIP^−/−^* lungs showed signs of vascular regression as demonstrated by large avascular areas (Fig. 4B,C). In addition, TUNEL (TdT-mediated dUTP-biotin nick end labeling) staining to detect apoptotic cells already showed some apoptotic capillary endothelial cells in E18.5 *KLEIP^−/−^* lungs but the percentage of apoptotic type II pneumocytes remained unaltered (Fig. 4D–F). After birth, cell apoptosis in *KLEIP^−/−^* lungs was still increased (Fig. 4E) but the maturation of larger lung vessels was normal (supplementary material Fig. S4).

**Fig. 5.**
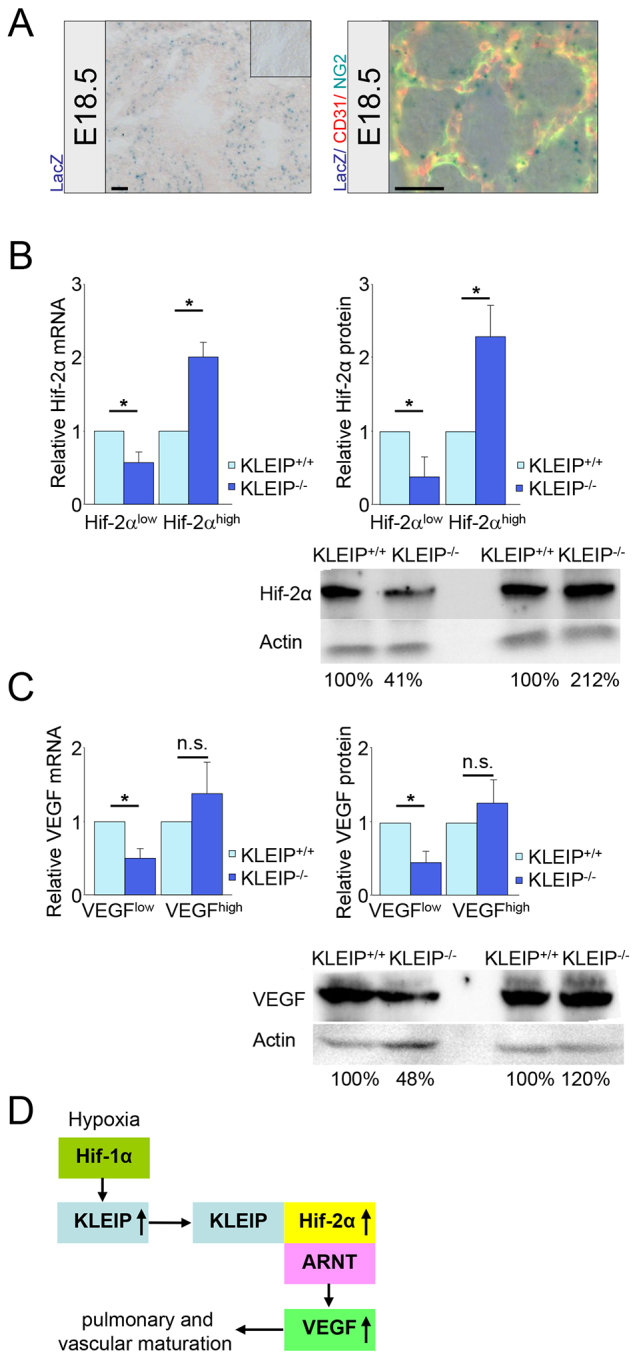
**KLEIP regulates expression of Hif-2α and VEGF in lung.** (A) Left: β-galactosidase (LacZ) staining in embryonic *KLEIP*^−/−^ lungs (E18.5) monitors *KLEIP* promoter activity; *KLEIP*^+/+^ lung sections served as controls (inset). Right: triple staining shows KLEIP expression (LacZ) in mesenchymal-derived pericytes (NG2, green) and in endothelial cells (CD31, red) of peripheral lung capillaries. Scale bars: 25 μm. (B) Expression analyses for *Hif-2α* mRNA (left) and Hif-2α protein (right) in E18.5 *KLEIP^+/+^* and *KLEIP^−/−^* lungs identifies two distinct populations. Hif-2α protein and mRNA expression increased 2.5-fold in 44% and 40%, respectively, of *KLEIP^−/−^* lungs (*KLEIP^−/−^* [Hif-2α^high^]; *n*=13 and 4, respectively), and in 56% and 60% of *KLEIP^−/−^* lungs Hif-2α protein and mRNA expression was decreased (*KLEIP^−/−^* [Hif-2α^low^]; *n*=18 and 6, respectively) (*KLEIP^+/+^*: protein *n*=32, mRNA *n*=6). Reduced expression of Hif-2α at E18.5 in 56% (protein) and 60% (mRNA) of all *KLEIP^−/−^* embryonic lungs reflects incidence for respiratory failure in neonatal *KLEIP^−/−^* mice (56% respiratory failure in *KLEIP^−/−^* newborns, Fig. 1D). Bottom right: representative western blot for E18.5 Hif-2α lung expression including its individual quantification. (C) Similar to Hif-2α expression, *VEGF* mRNA (left) and VEGF_164_ protein (right) expression analyses identifies two distinct groups. VEGF protein and mRNA expression was weakly increased in 41% and 44%, respectively, of E18.5 *KLEIP^−/−^* lungs (*KLEIP^−/−^* [VEGF^high^]; *n*=11 and 4, respectively), and in 59% and 56% of E18.5 *KLEIP^−/−^* embryos VEGF protein and mRNA was decreased (*KLEIP^−/−^* [VEGF^low^]; *n*=16 and 6, respectively) (*KLEIP^+/+^*: protein *n*=29, mRNA *n*=6). Bottom right: representative western blot for E18.5 VEGF_164_ lung expression including its individual quantification. (D) Proposed model for KLEIP’s function in lung maturation. KLEIP is upregulated in lung capillaries by hypoxia in a Hif-1α-dependent manner. KLEIP binds to Hif-2α and thereby increases Hif-2α stability and Hif-2α transcriptional activation, which promotes VEGF expression, leading to pulmonary vascular remodeling and late-stage embryonic lung maturation. n.s.: not significant, *P<0.05.

In order to characterize KLEIP expression in lung, stainings for β-galactosidase (*lacZ*), which was under control of the *KLEIP* promoter, of E18.5 *KLEIP^−/−^* lungs were performed. During the saccular stage, β-galactosidase staining showed KLEIP expression in lung endothelium and in pericytes (Fig. 5A). Interestingly, Hif-2α, which is transcriptionally activated by KLEIP (Higashimura et al., 2011), is also expressed in lung endothelial cells (Skuli et al., 2009; Wagner et al., 2004). However, neither double staining for β-galactosidase and SP-C nor for β-galactosidase and E-cadherin were positive, indicating KLEIP absence in the lung epithelium (supplementary material Fig. S5). This is in contrast to Hif-2α expression, which is also highly present in developing lung epithelial cells (Wagner et al., 2004). This observation led to the conclusion that KLEIP acts as a hypoxia-inducible factor in lung capillaries and that KLEIP regulates blood vessel function as shown by increased endothelial cell apoptosis in *KLEIP^−/−^* lungs (Fig. 4D). It further suggested a paracrine function for KLEIP on pneumocyte maturation as evidenced by insufficient surfactant production by type II pneumocytes (Fig. 3C). This is underlined by data showing that vascular remodeling and pneumocyte differentiation are key processes for late-stage lung maturation and both processes are regulated by Hif-1α, Hif-2α and VEGF (Akeson et al., 2005; Akeson et al., 2003; Compernolle et al., 2002; Del Moral et al., 2006; Saini et al., 2008; Wagner et al., 2004).

Because KLEIP is regulated by hypoxia in a Hif-1α-dependent manner (Nacak et al., 2007; Yuan et al., 2011) and Hif-2α binds to and is transcriptionally activated by KLEIP (Higashimura et al., 2011), we next analyzed the relationship between KLEIP and Hif-2α expression in embryonic *KLEIP^+/+^* and *KLEIP^−/−^* lungs. Protein and mRNA expression analyses for Hif-2α in E18.5 *KLEIP^−/−^* lungs identified two groups. In ~40% of embryonic *KLEIP^−/−^* lungs a twofold upregulation of *Hif-2α* mRNA and protein was identified, whereas ~60% of embryonic *KLEIP^−/−^* lungs showed a significant downregulation of *Hif-2α* mRNA and protein (Fig. 5B). Strikingly, reduced expression at E18.5 for Hif-2α in ~60% of all *KLEIP^−/−^* lungs exactly reflects the incidence for respiratory failure in neonatal *KLEIP^−/−^* mice (respiratory failure in 56% of *KLEIP^−/−^* neonates, Fig. 1D). Therefore, reduced embryonic Hif-2α expression in *KLEIP^−/−^* lungs at E18.5 suggests Hif-2α as a predetermination factor for the development of respiratory failure after birth. Because neonatal *KLEIP^−/−^* mice suffer from vascular dysfunction (Fig. 4B) and Hif-2α regulates VEGF expression, which is an important maturation factor for pneumocyte differentiation, we finally addressed the question of whether VEGF could be a target of the KLEIP–Hif-2α signaling cascade in embryonic lungs. To this end, VEGF mRNA and protein analyses in embryonic *KLEIP^−/−^* lungs were performed. It was found that VEGF expression was compensated in those embryonic *KLEIP^−/−^* lungs that showed enhanced Hif-2α expression and resembled VEGF levels as were observed in *KLEIP^+/+^* mice (Fig. 5C). In sharp contrast, embryonic *KLEIP^−/−^* lungs that displayed reduced Hif-2α expression also showed significantly reduced VEGF mRNA and protein expression (Fig. 5C). Likewise, protein expression for SP-C in *KLEIP*^−/−^ lungs showed similarity to Hif-2α expression within two distinct groups. In 40% of E18.5 *KLEIP*^−/−^ lungs SP-C expression was increased, whereas in 60% of E18.5 *KLEIP*^−/−^ lungs SP-C protein expression was reduced (supplementary material Fig. S6). In conclusion, the data demonstrate that KLEIP deficiency can be rescued by about 40% of the animals by compensatory Hif-2α and VEGF expression, whereas about 60% of *KLEIP^−/−^* animals are unable to do owing to a reduction of Hif-2α expression within them. Thus, KLEIP increases VEGF expression via Hif-2α stabilization and transcriptional activation, which finally promotes vascular and pulmonary development (Fig. 5D).

### Glucocorticoid therapy compensates respiratory distress in *KLEIP^−/−^* mice

**Fig. 6.**
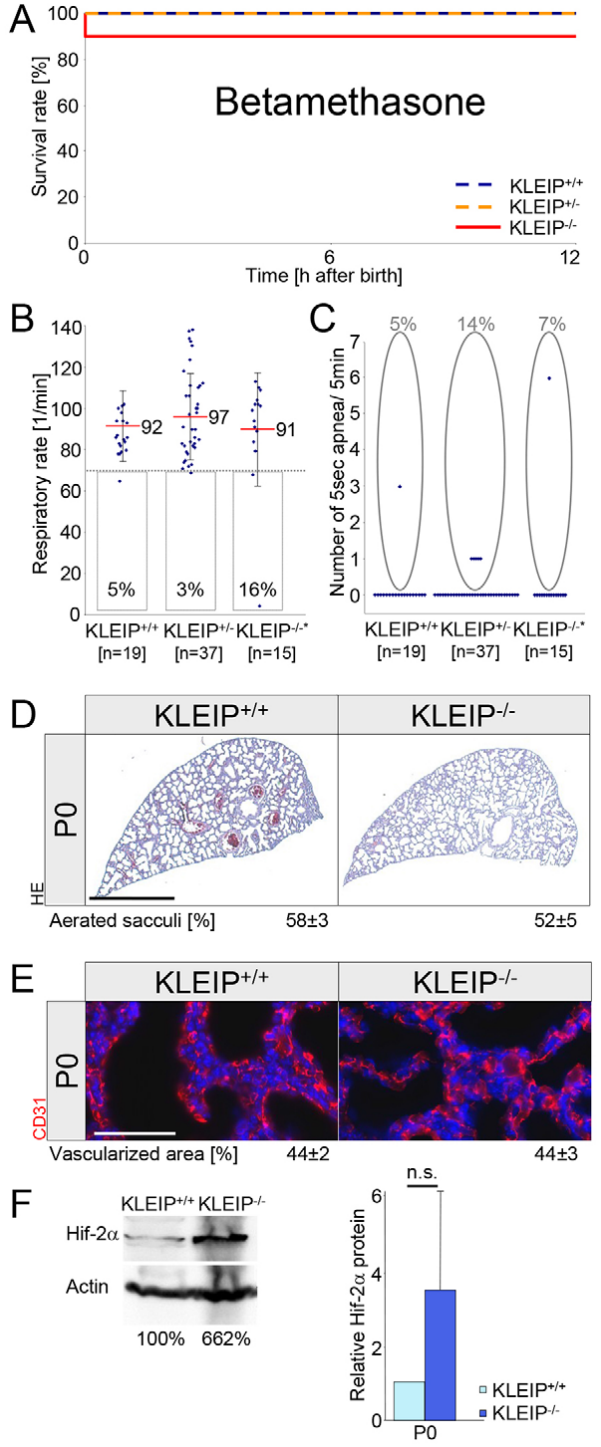
**Betamethasone prevents respiratory failure in *KLEIP^−/−^* neonates.** (A) Survival curve for *KLEIP^+/+^*, *KLEIP^+/−^* and *KLEIP^−/−^* neonates after embryonic betamethasone treatment. Whereas 12% of untreated *KLEIP^+/−^* and 38% of untreated *KLEIP^−/−^* newborns die after birth (Fig. 1B), 88% of betamethasone-treated *KLEIP^−/−^* (*n*=16) and 100% of betamethasone-treated *KLEIP^+/−^* (*n*=36) mice survive. *KLEIP*^+/+^ (*n*=23) littermates served as controls. (B) Injection of betamethasone into pregnant *KLEIP^+/−^* mothers increases respiratory rates of *KLEIP^−/−^* neonates. Betamethasone-treated *KLEIP^+/+^*, *KLEIP^+/−^* and *KLEIP^−/−^* mice breathe on average 92 minute^−1^, 97 minute^−1^ or 91 minute^−1^, respectively. (C) Apnea were recorded for 5% of *KLEIP^+/+^*, 14% of *KLEIP^+/−^* and 7% of *KLEIP^−/−^* betamethasone-treated newborns. * indicates two dead *KLEIP^−/−^* neonates that were not included into the respiratory statistics. (D) Histological staining for HE demonstrates physiological aerated *KLEIP^−/−^* lungs after betamethasone treatment. *KLEIP^+/+^* and *KLEIP^−/−^* newborns show an equal, homogenous aeration of lung sacculi 12 hours after birth. Scale bar: 1 mm. Quantification: *n*=5 mice per group. HE: haemalaun-eosin. (E) Betamethasone treatment maintains lung capillary network in *KLEIP^−/−^* neonates. The capillary network was analyzed by anti-CD31 staining. The staining revealed a comparable, intact capillary network in *KLEIP^+/+^* and *KLEIP^−/−^* newborns. Scale bar: 50 μm. Quantification: *n*=5 mice per group. (F) Betamethasone treatment at E18.5 and E19.5 normalizes/increases Hif-2α expression in P0 *KLEIP*^−/−^ lungs as compared with *KLEIP*^+/+^ littermates. Left: representative western blots including its individual quantification; right: quantification of all western blots for *n*=5 lungs per group. n.s.: not significant.

In humans, pregnant mothers at increased risk of preterm delivery are treated with glucocorticoids to support lung maturation and to prevent respiratory distress. Glucocorticoids can increase VEGF expression in mice (Bhatt et al., 2000) and in rat (San Feliciano et al., 2011). Based on these data we hypothesized a rescue of respiratory failure in *KLEIP^−/−^* neonates through betamethasone injection. Therefore, we injected betamethasone on E18.5 and E19.5 in *KLEIP^+/−^* pregnant mice and observed an increased survival rate and reduced cyanosis in *KLEIP^+/−^* and *KLEIP^−/−^* neonates. Neonatal lethality decreased in *KLEIP^−/−^* mice from 38% to 12% and in *KLEIP^+/−^* neonates from 12% to 0% (Fig. 6A versus Fig. 1B). Analysis of respiratory rates revealed normal respiration and reduced apnea in almost all treated *KLEIP^+/−^* and *KLEIP^−/−^* neonates (Fig. 6B,C versus Fig. 1C,D). These findings are complemented by normal lung development and aeration (Fig. 6D) and physiological lung capillarization (Fig. 6E) in *KLEIP^−/−^* neonates. Finally, we studied the effect of betamethasone on Hif-2α expression in endothelial cells and in P0 *KLEIP*^+/+^ and *KLEIP*^−/−^ lungs. Under normoxic conditions, endothelial Hif-2α expression was very low and could not be increased by betamethasone (supplementary material Fig. S7A). However, under hypoxic conditions endothelial Hif-2α expression was increased, which was strongly amplified after betamethasone treatment (supplementary material Fig. S7A). In *KLEIP*^−/−^ neonates, betamethasone treatment enhanced lung Hif-2α expression, which reached even higher levels than in betamethasone-treated *KLEIP*^+/+^ littermates (Fig. 6F). Likewise, betamethasone prevented a significant increase of endothelial cell apoptosis in *KLEIP*^−/−^ neonates (supplementary material Fig. S7B). Together, these important findings identify endothelial Hif-2α as a newly identified target of betamethasone, disclosing an important step for betamethasone’s mechanism of action, which, until now has not been described. Thus, betamethasone increases vascular stability and lung maturation through upregulation of Hif-2α, which finally prevents respiratory failure in *KLEIP^−/−^* mice.

## DISCUSSION

In this study, we define a newly identified functional interaction between KLEIP, Hif-2α and VEGF in late-stage embryonic lung development in mice and we identified betamethasone, commonly used in clinical RDS prevention, as a positive regulator of this newly characterized signaling cascade. Within more than half of the *KLEIP^−/−^* mice, KLEIP deficiency leads to epithelial immaturity and neonatal respiratory failure. Subsequently, several *KLEIP^−/−^* neonates died shortly after birth. *KLEIP^−/−^* neonates with respiratory failure showed reduced lung aeration and increased septum thickness. Embryonic expression analyses and rescue experiments with betamethasone identified the KLEIP–Hif-2α–VEGF axis as a predetermination factor for postnatal respiratory failure, functioning in an angiocrine manner on pneumocytes.

The vascular system is usually considered as an organ that facilitates the transport of gases, nutrients and waste products, and regulates immune responses. Yet, recent data suggested an active, paracrine function of capillary endothelial cells on epithelial cell differentiation during organ development and regeneration, and the term ‘angiocrine function’ has been established (Butler et al., 2010a; Butler et al., 2010b). In this scenario, blood-vessel-derived angiocrine factors play a key role in orchestrating development and regeneration of the liver (Ding et al., 2014; Ding et al., 2010; Hu et al., 2014; Matsumoto et al., 2001). Likewise, regenerative lung growth is linked to angiocrine signaling (Ding et al., 2011). The fact that pulmonary vascular development and airway branching are interdependent also suggested an angiocrine function in lung organogenesis (Gebb and Shannon, 2000; van Tuyl et al., 2005). In *KLEIP^−/−^* mice, embryonic development including vasculogenesis and angiogenesis is generally normal. Yet, at E18.5 a clear difference could be observed in *KLEIP^−/−^* lungs, showing epithelial immaturity. Premature E18.5 *KLEIP^−/−^* lungs showed reduced type II pneumocyte differentiation, resulting in insufficient surfactant production. In addition, embryonic *KLEIP^−/−^* lungs displayed normal glycogen accumulation but, after birth, reduced transformation of glycogen into surfactant was observed, which is known to cause severe RDS and neonatal death (Whitsett et al., 2004). Although *KLEIP^−/−^* lungs displayed few peripheral bleedings and some apoptotic endothelial cells, the vascular system in E18.5 *KLEIP^−/−^* lungs appeared largely functional because blood vessels were filled with blood and capillary density was unaltered. Therefore, the strong epithelial phenotype in *KLEIP^−/−^* lungs is likely not based on reduced oxygen and nutrient supply because, before E18.5, lung epithelial development was normal. This conclusion is further supported by data from the conditional endothelial-specific Hif-2α mouse, in which minor lung vascular defects did not affect Mendelian ratios of born mice and no obvious developmental defects could be observed (Skuli et al., 2009). Thus, the data show an essential function for KLEIP in late-stage lung maturation and suggest pneumocyte immaturity and not vascular damage to be the reason for neonatal respiratory failure in *KLEIP*^−/−^ neonates.

KLEIP has originally been identified as a hypoxia-inducible protein *in vitro* that regulates the stability and transcriptional activity of Hif-2α, which suggested a functional relationship between KLEIP and Hif-2α (Higashimura et al., 2011; Nacak et al., 2007). Our study identified a new functional interaction during late-stage lung development *in vivo* because Hif-2α expression was strongly altered in E18.5 *KLEIP^−/−^* embryos. Remarkably, two groups for Hif-2α expression could be identified. Given the fact that KLEIP stabilizes and activates Hif-2α (Higashimura et al., 2011), KLEIP deficiency in mice should result in decreased Hif-2α expression. Indeed this was found in more than half of the E18.5 *KLEIP^−/−^* lungs. After birth, the same number of *KLEIP^−/−^* newborns (56%) suffered from respiratory failure, which denotes that the KLEIP–Hif-2α axis acts before birth as a late-stage maturation factor for normal lung development and that embryonic KLEIP–Hif-2α levels determine whether neonates suffer from respiratory failure or not. It also defines a certain threshold for VEGF that is necessary for physiological lung development that needs to be produced by the lung itself and cannot be adequately compensated by systemic VEGF. This is further supported by the fact that about 40% of E18.5 *KLEIP^−/−^* embryos are able to compensate, by an unknown mechanism, for KLEIP deficiency, leading to increased Hif-2α levels, physiological VEGF concentrations and, subsequently, to neonatal *KLEIP^−/−^* mice that develop normally.

VEGF is a key regulator of vascular development and homeostasis. However, during the last decade an important function for VEGF in embryonic pulmonary epithelial maturation has been described (Akeson et al., 2005; Akeson et al., 2003; Compernolle et al., 2002; Del Moral et al., 2006). In the lungs, VEGF acts via VEGF receptor 2 on pneumocytes, and several E18.5 *KLEIP^−/−^* lungs showed reduced VEGF levels, indicating reduced embryonic lung maturation, e.g. reduced surfactant production caused by insufficient VEGF supply. Expression analyses for KLEIP in embryonic lungs showed its abundance in blood vessels but not in epithelial cells, whereas Hif-2α is expressed in both cell types (Wagner et al., 2004). Therefore, reduced expression of capillary VEGF in E18.5 *KLEIP^−/−^* lungs is caused by KLEIP absence, which induces destabilization and transcriptional inactivation of Hif-2α, leading to reduced VEGF expression. Subsequently, reduced vascular VEGF expression abrogates angiocrine VEGF signaling, leading to dysfunctional type II pneumocytes. Thus, expression levels of KLEIP, Hif-2α and VEGF in embryonic lungs determine whether respiratory failure develops after birth. Importantly, standard glucocorticoid treatment using betamethasone in *KLEIP^−/−^* embryos almost completely rescued respiratory failure in *KLEIP^−/−^* newborns. Betamethasone strongly increases Hif-2α expression in cultured endothelial cells and *KLEIP*^−/−^ neonatal lungs. Therefore, this study shows that betamethasone acts on this newly identified signaling cascade during late-stage lung maturation. Thus, the data identified a previously unknown mechanism of glucocorticoid action through endothelial Hif-2α.

Collectively, this study identified the KLEIP–Hif-2α axis as a new target for betamethasone, which rescues respiratory failure in *KLEIP^−/−^* neonates, and shows two distinct functions for KLEIP in late-stage lung development: firstly, a moderate autocrine function for KLEIP regulating maintenance of embryonic lung endothelial cells and, secondly, a strong angiocrine function of lung capillaries on epithelial pneumocytes via Hif-2α and VEGF (Fig. 5D). Because KLEIP is not expressed in the lung epithelium but regulates VEGF expression, our findings support the concept that late-stage embryonic pulmonary epithelial maturation is determined by KLEIP-dependent angiocrine Hif-2α–VEGF signaling.

## MATERIALS AND METHODS

### *B6.129-KLEIP^tm/Mhm^* transgenic mice and therapeutical studies

Generation and genotyping of *KLEIP^−/−^* mice has been previously described (Hahn et al., 2012). Animal housing and animal studies were approved by the Regierungspräsidium Karlsruhe. For therapeutic interventions, 0.4 mg/kg body weight CELESTAN^®^ (MSD Sharp & Dohme GmbH) was administered by intramuscular (i.m.) injection into *KLEIP^+/−^* pregnant females at E18.5 and E19.5. This protocol is based on a recent study in rat (San Feliciano et al., 2011) in which injection of betamethasone was performed twice in pregnant rats, at 1 and 2 days before birth.

### Cell culture experiments

Human umbilical vein endothelial cells (HUVECs) were isolated and cultured as previously described (Stoll et al., 2011). Cells were incubated with 0.4 mg/l CELESTAN^®^ for 24 hours under normoxic (21% O_2_) or hypoxic (0.6% O_2_) conditions. Protein lysates were investigated by western blot analysis.

### Antibodies and histological reagents

The following antibodies and kits were used for immunohistochemistry and western blot: anti-mouse CD31, clone SZ31, Dianova; anti-human/mouse NG2, Millipore; anti-mouse E-cadherin, clone ECCD-2, Life Technologies; anti-human SMA, clone 1A4, Sigma-Aldrich; anti-human Ki67, Leica Biosystems; anti-mouse CC10, Abcam; anti-mouse proSP-C, Millipore; anti-human Hif-2α, Novus Biologicals; anti-mouse VEGF (A-20), Santa Cruz Biotechnology; anti-mouse actin, Santa Cruz Biotechnology; anti-rabbit HRP, DAKO; anti-rat Alexa Fluor 546, Molecular Probes; anti-rabbit Alexa Fluor 488, Molecular Probes; anti-rabbit FITC, Dianova; HRP-conjugated antibodies and ABC-kit, VECTOR Laboratories; DAPI, Sigma-Aldrich; haemalaun-eosin (HE) staining solution, Sigma-Aldrich; Trichrome Stain (Masson) Kit, Sigma-Aldrich; PAS Kit, Sigma-Aldrich.

### Immunhistochemistry

Tissues from *KLEIP^+/+^* and *KLEIP^−/−^* neonates were prepared and fixed in 4% formaldehyde overnight. Fixed lungs were embedded in OCT-medium or dehydrated (70%, 80%, 90%, 100% ethanol, 100% xylol, 60 minutes each) and embedded in paraffin. Blocks were cut to a size of 8 μm. For immunostaining, paraffin sections were rehydrated using reverse ethanol series and cooked 30 minutes in citrate buffer (10 mM citrate, pH 6.0); cryosections were hydrated in PBS. Sections were permeabilized and washed in PBS + 0.5% Tween-20 (PBST), blocked in 5% goat serum and embedded in Dako mounting medium (Invitrogen). For HE staining, slides were deparaffinized using reverse ethanol series, incubated for 1 minute in hematoxylin, rinsed continuously under tap water, stained for 1 minute with eosin solution, rinsed in tap water, dehydrated using ethanol series and mounted in DPX. Apoptotic lung cells were labeled using the TUNEL staining kit from Chemicon/Millipore (ApopTag^®^ Red *In Situ* Apoptosis Detection Kit) and PAS staining was conducted using the Periodic Acid-Schiff Kit according to the manufacturer’s protocol (Sigma-Aldrich).

### β-galactosidase (*lacZ*) staining

β-galactosidase staining was performed as recently described (Hahn et al., 2012; Lobe et al., 1999). Briefly, tissues were fixed in LacZ-fix solution containing 0.2% glutaraldehyde at 4°C overnight, washed three times in PBS, embedded in OCT-medium and cut to 8-μm tissue sections. Tissue sections were washed three times in LacZ wash buffer, and incubated overnight at 37°C in β-galactosidase stain containing 1 mg/ml X-Gal (BIOMOL prod. no. 02249, Hamburg, Germany), 2.1 mg/ml potassium ferrocyanide and 1.6 mg/ml potassium ferricyanide. 16 hours later tissue sections were washed three times in PBS, post-fixed in 2% PFA/0.1% glutaraldehyde/PBS and further processed for immunohistochemistry.

### RNA isolation and quantitative PCR

Lungs were mechanically homogenized. Total mRNA was isolated using the RNeasy™ Mini Kit (Qiagen) and RT-PCR was generated by using Superscript II RT™ (Promega). Quantitative real-time PCR was performed using the PowerSYBR Green PCR Master Mix (Life Technologies) under the following conditions: 95°C for 10 minutes; cycling (35×): 95°C for 30 seconds, 60°C for 30 seconds, 72°C for 30 seconds; melting curve: 95°C for 1 minute, 55°C for 30 seconds. The following primers were used: *KLEIP*: forward 5′-TGGCACAACATACTTGAAGAC-3′ (binds Exon 10); reverse 5′-AATAACTCCTACACCACCTCC-3′ (binds Exon 11); *Hif-2α*: forward 5′-CCTGGCCATCAGCTT-3′ (binds Exon 2); reverse 5′-CTGGTCGGCCTCAGCTTC-3′ (binds Exon 3); *VEGF*: forward 5′-GAGTACCCCGACGAGATAGAGT-3′ (binds Exon 3); reverse 5′-GGTGAGGTTTGATCCGCATGA-3′ (binds Exon 4); *GAPDH*: forward 5′-CTGCACCACCAACTGCTTAG-3′ (binds Exon 4); reverse 5′-TGGATGCAGGGATGATGTTC-3′ (binds Exon 5); *SP-A*: forward 5′-GGCAGACATCCACACAGCCT-3′ (binds Exon 1); reverse 5′-ACTTGATGCCAGCAACAACAGT-3′ (binds Exon 3); *SP-B*: forward 5′-ACAAGGCCCTCAATTCTGGTGC-3′ (binds Exon 2); reverse 5′-CAGGTCATTAGCTCCTGCATGC-3′ (binds Exon 2 and 3); *SP-C*: forward 5′-ATGAGTAGCAAAGAGGTCCTG-3′ (binds Exon 1); reverse 5′-TGGTGTCTGCTCGCTCACTC-3′ (binds Exon 3); *SP-D*: forward 5′-AGAGCCTCTCGCAGAGATCAG-3′ (binds Exon 1); reverse 5′-GAGGTCCACTTAGTCCACGTTC-3′ (binds Exon 1 and 2).

### Western blot analysis

Cells were directly lysed, whereas tissues were homogenized by pestle and syringe and afterwards lysed in buffer (150 mmol/l NaCl, 50 mmol/l Tris-HCl, pH 7.4, 1% NP40, 10 mmol/l EDTA, 10% glycerol, and protease inhibitors). Protein concentration was detected by BCA-Assay (Thermo Scientific). Equal amounts of protein were separated by 10% SDS-PAGE and blotted to nitrocellulose membrane (Sigma-Aldrich). Blots were incubated with the indicated antibodies. Finally, signals were visualized using the ECL reagent (Perbio Science). Densitometric quantification of the signals was performed using the Gel-Pro Analyzer 6.0 software (Media Cybernetics) and normalized to β-actin.

### Quantification and statistical analysis

Histological sections were analyzed with a Zeiss Axio Imager D1 or Z1 fluorescence microscope (Carl Zeiss) and Zeiss AxioVision software. For quantification of aerated sacculi, the number of lung sacculi was determined for one complete lung cross-section per animal using ImageJ software. Sacculi bigger than 100 μm^2^ were considered as aerated. The ratio of aerated sacculi to total sacculi was compared within the different individuals. *n*≥4 *KLEIP^+/+^* and *KLEIP^−/−^* embryos and neonates were investigated. Septum thickness was assessed within at least 5 power fields. All septa within this area were measured using Zeiss AxioVision software. The average septum thickness was compared between *KLEIP^+/+^* and *KLEIP^−/−^* embryos or neonates, respectively (*n*≥4 mice per group). For quantification of PAS-, SP-C- and CC10-positive cells, HE-positive tissue area was considered as total lung tissue area. For each animal, PAS-positive cells were individually counted (*n*=5 animals per group and 5 power fields for each *KLEIP^+/+^* and *KLEIP^−/−^* embryo/neonate). Number of cells positive for SP-C were counted using ImageJ and set in relation to the total lung tissue area. CC10-positive tissue area was determined using ImageJ and set in relation to total tissue area. For SP-C and CC10 stainings, quantification was done for *n*=5 animals per group and 3 power fields (one complete cross-section for CC10) for each animal (*KLEIP^+/+^* and *KLEIP^−/−^* embryos and neonates). To determine lung vascularization, DAPI-positive total tissue area and CD31-positive tissue area were determined using ImageJ, and CD31-positive areas were put in relation and compared between *KLEIP^+/+^* and *KLEIP^−/−^* embryos and neonates (*n*=5 mice per group and 5 power fields for each animal). Apoptotic cells in one complete lung cross-section were counted and set in relation to the total lung area using ImageJ. *n*≥6 animals per group of *KLEIP^+/+^* and *KLEIP^−/−^* embryos and neonates were comparatively analyzed. The rate of apoptotic type II pneumocytes and endothelial cells in relation to total apoptotic cells (*n*=5 animal and 5 power fields) was determined by ImageJ. Results are shown as means ± s.d. Comparisons between groups were analyzed by Student’s *t*-test or Mann-Whitney *U* test using SPSS software (IBM). *P*<0.05 was considered as statistically significant.

## Supplementary Material

Supplementary Material
