# Supplementary material for: Kelch-like ECT2-interacting protein KLEIP regulates late-stage pulmonary maturation via Hif-2α in mice
Source: Dis Model Mech. 2014 May 1;7(6):683–92. doi: 10.1242/dmm.014266 (PMC4036475; doi:10.1242/dmm.014266)
Supplement: Supplementary Material [file supp_7_6_683__index.html]

Kelch-like ECT2-interacting protein KLEIP regulates late-stage pulmonary maturation via Hif-2α in mice — Supplementary Material 

# Kelch-like ECT2-interacting protein KLEIP regulates late-stage pulmonary maturation via Hif-2α in mice

## DMM014266 Supplementary Material

**Files in this Data Supplement:**

- **Supplementary Material**
