# Supplementary material for: Kelch-like ECT2-interacting protein KLEIP regulates late-stage pulmonary maturation via Hif-2α in mice
Source: Dis Model Mech. 2014 May 1;7(6):683–92. doi: 10.1242/dmm.014266 (PMC4036475; doi:10.1242/dmm.014266)
Supplement: Supplementary Material [file supp_7.6.683_DMM014266.pdf]

Supplemental Information

Kelch-like ECT2 interacting protein KLEIP regulates late stage pulmonary maturation via Hif-2α

Nicole Woik[1,2], Christian T. Dietz[1], Kathrin Schäker[1,2] and Jens Kroll[1,2,#]

[1]Department of Vascular Biology and Tumor Angiogenesis, Center for Biomedicine and Medical Technology Mannheim (CBTM), Medical Faculty Mannheim of Heidelberg University, Mannheim, Germany and [2]Division of Vascular Oncology and Metastasis, German Cancer Research Center (DKFZ-ZMBH Alliance), Heidelberg, Germany

Running title: KLEIP in respiratory failure

[#]Correspondence should be addressed to:
Dr. Jens Kroll
Center for Biomedicine and Medical Technology Mannheim (CBTM)
Dept. of Vascular Biology and Tumor Angiogenesis, Medical Faculty Mannheim of Heidelberg University
Ludolf-Krehl-Str. 13-17
68167 Mannheim, Germany
Phone: +49-(0)621-383-9965
Fax: +49-(0)621-383-9961
Email: jens.kroll@medma.uni-heidelberg.de
www.angiolab.de

Supplemental Figure legends:

Suppl. Fig. 1: Survival statistics for *KLEIP*$^{-/-}$ mice

(A) Mendel's distribution at E18.5 (left panel) shows slightly reduced numbers for *KLEIP*$^{-/-}$ embryos (22%). At P0.5 (up to 12h after birth) only 15% of *KLEIP*$^{-/-}$ pups are still alive (middle panel) which does not significantly change until P28 (13% of *KLEIP*$^{-/-}$ mice are alive, right panel). n=32 embryos at E18.5; 151 pups at P0.5 and 225 mice at P28. (B) Lungs of newborn *KLEIP*$^{-/-}$ mice with severe respiratory failure sank to bottom while lungs of *KLEIP*$^{+/+}$ littermates floated in PBS solution indicating missing aeration in *KLEIP*$^{-/-}$ lungs.

Suppl. Fig. 2: Few *KLEIP*$^{-/-}$ embryos die due to embryonic bleedings

Representative image showing embryonic bleedings and growth retardation in an E12.5 *KLEIP*$^{-/-}$ embryo. Scale bar: 1mm.

Suppl. Fig. 3: *KLEIP*$^{-/-}$ mice die not due to diaphragmic hernia, airway closure and growth retardation

(A) Morphological characterization of *KLEIP*$^{-/-}$ diaphragms at P0 did not indicate morphological alteration compared to *KLEIP*$^{+/+}$ diaphragms. (B) Morphological analyses of *KLEIP*$^{-/-}$ pups at P0 did not reveal morphological defects in secondary palate formation as a potential reason for respiratory failure (d, dorsal; v, ventral). (C) Relative body weight of *KLEIP*$^{+/+}$, *KLEIP*$^{+/-}$ and *KLEIP*$^{-/-}$ mice at E18.5 (n=7:20:4), P0 (n=6:13:4), P28 (n=13:14:5) and 10 weeks (n=21:11:20). (D) Quantification of type II pneumocytes (left panel) and Clara cells (right panel) at E18.5 and P0 revealed no significant alterations in cell number in *KLEIP*$^{-/-}$ embryos and neonates (n= 5 mice per group). n.s.: not significant. Scale bars: 1mm.

Suppl. Fig. 4: Normal development of large blood vessels and bronchi in *KLEIP*$^{-/-}$ lungs

αSMA as a smooth muscle cell marker indicates at P0 physiological maturation of large blood vessels (asterisks) and bronchia (points) in *KLEIP*$^{-/-}$ and KLEIP$^{+/+}$ lungs. Scale bar: 100µm.

Suppl. Fig. 5: KLEIP is not expressed in lung epithelium

(A) LacZ positive cells as an indicator for KLEIP expression do not colocalize with SP-C or (B) E-Cadherin in E18.5 *KLEIP*$^{-/-}$ lungs.

Suppl. Fig. 6: SP-C protein expression at E18.5 in KLEIP$^{-/-}$ and KLEIP$^{+/+}$ lungs shows two distinct groups. In 60% of KLEIP$^{-/-}$ lungs (n=6) SP-C protein was decreased while in 40% of KLEIP$^{-/-}$ lungs (n=4) SP-C protein expression was increased. This correlates with lung Hif-2α expression at E18.5 as shown in Fig. 5B. Left: Representative Western blot including its individual quantification. *p<0.05.

Suppl. Fig. 7: Betamethasone increases endothelial Hif-2α expression and prevents endothelial apoptosis in P0 KLEIP$^{-/-}$ lungs.

(A) Betamethasone treatment stimulates Hif-2α expression under hypoxic conditions in cultured endothelial cells. The effect of betamethasone on HUVEC was investigated under normoxic (21%O_2) and hypoxic (0.6%O_2) conditions. While betamethasone did not increase Hif-2α synthesis under normoxic conditions, it strongly enhanced Hif-2α expression under hypoxia; n=3. (B) Quantification of TUNEL/CD31 double positives cells in relation to total TUNEL positive cells after betamethasone treatment in P0 KLEIP$^{-/-}$ lungs did not show significant increased apoptotic rates (n=5 animals per group). n.s.: not significant.

Suppl. Fig. 1, N. Woik et al., 2014

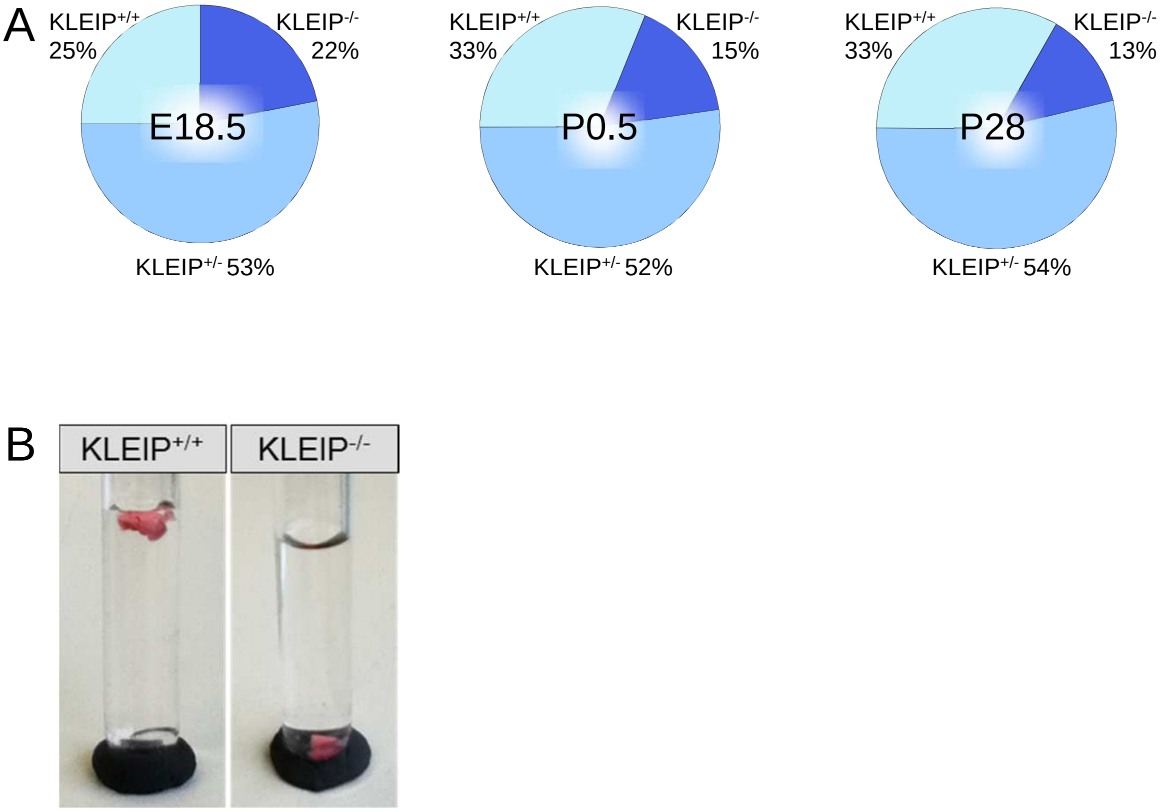

Suppl. Fig. 1: Survival statistics for *KLEIP*$^{-/-}$ mice. (A) Mendel's distribution at E18.5 (left panel) shows slightly reduced numbers for *KLEIP*$^{-/-}$ embryos (22%). At P0.5 (up to 12h after birth) only 15% of *KLEIP*$^{-/-}$ pups are still alive (middle panel) which does not significantly change until P28 (13% of *KLEIP*$^{-/-}$ mice are alive, right panel). n=32 embryos at E18.5; 151 pups at P0.5 and 225 mice at P28. (B) Lungs of newborn *KLEIP*$^{-/-}$ mice with severe respiratory failure sank to bottom while lungs of *KLEIP*$^{+/+}$ littermates floated in PBS solution indicating missing aeration in *KLEIP*$^{-/-}$ lungs.

Suppl. Fig. 2, N. Woik et al., 2014

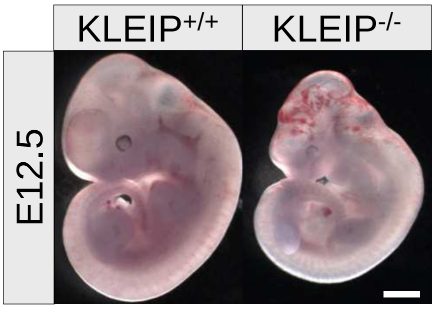

Suppl. Fig. 2: Few *KLEIP*$^{-/-}$ embryos die due to embryonic bleedings. Representative image showing embryonic bleedings and growth retardation in an E12.5 *KLEIP*$^{-/-}$ embryo. Scale bar: 1mm.

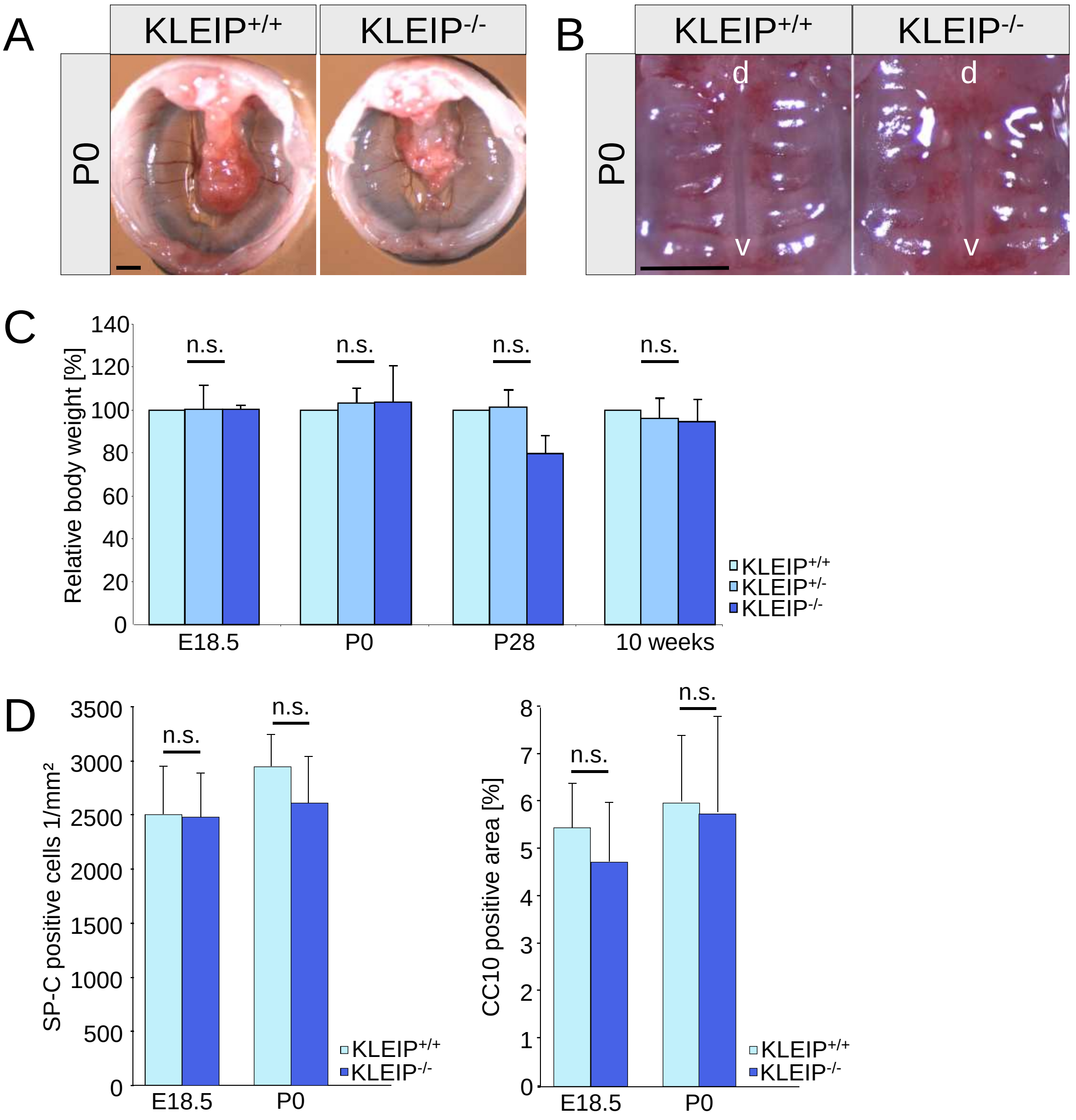

Suppl. Fig. 3: *KLEIP*$^{-/-}$ mice die not due to diaphragmic hernia, airway closure and growth retardation. (A) Morphological characterization of *KLEIP*$^{-/-}$ diaphragms at P0 did not indicate morphological alteration compared to *KLEIP*$^{+/+}$ diaphragms. (B) Morphological analyses of *KLEIP*$^{-/-}$ pups at P0 did not reveal morphological defects in secondary palate formation as a potential reason for respiratory failure (d, dorsal; v, ventral). (C) Relative body weight of *KLEIP*$^{+/+}$, *KLEIP*$^{+/-}$ and *KLEIP*$^{-/-}$ mice at E18.5 (n=7:20:4), P0 (n=6:13:4), P28 (n=13:14:5) and 10 weeks (n=21:11:20). (D) Quantification of type II pneumocytes (left panel) and Clara cells (right panel) at E18.5 and P0 revealed no significant alterations in cell number in *KLEIP*$^{-/-}$ embryos and neonates (n= 5 mice per group). n.s.: not significant. Scale bars: 1mm.

Suppl. Fig. 4, N. Woik et al., 2014

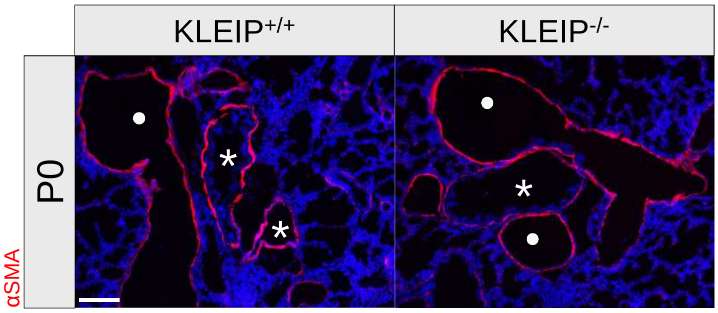

Suppl. Fig. 4: Normal development of large blood vessels and bronchi in *KLEIP*$^{-/-}$ lungs. αSMA as a smooth muscle cell marker indicates at P0 physiological maturation of large blood vessels (asterisks) and bronchia (points) in *KLEIP*$^{-/-}$ and KLEIP$^{+/+}$ lungs. Scale bar: 100µm.

Suppl. Fig. 5, N. Woik et al., 2014

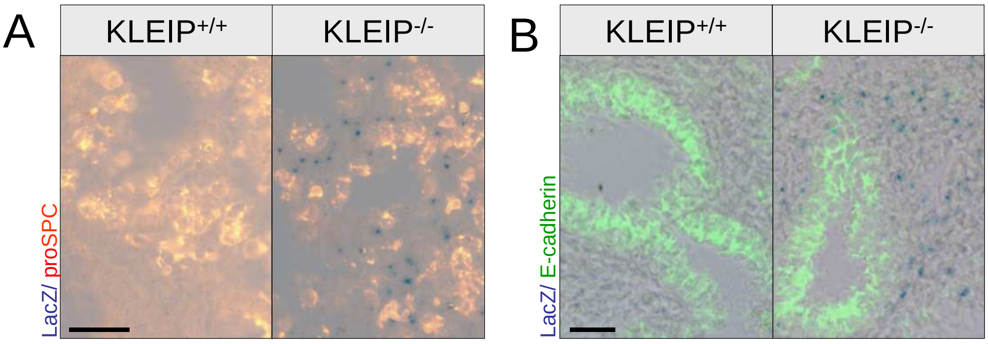

Suppl. Fig. 5: KLEIP is not expressed in lung epithelium. (A) LacZ positive cells as an indicator for KLEIP expression do not colocalize with SP-C or (B) E-Cadherin in E18.5 *KLEIP*$^{-/-}$ lungs.

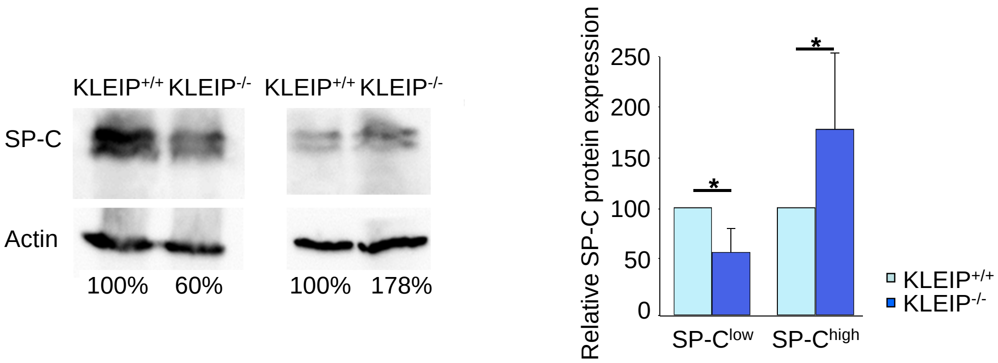

Suppl. Fig. 6: SP-C protein expression at E18.5 in KLEIP$^{-/-}$ and KLEIP$^{+/+}$ lungs shows two distinct groups. In 60% of KLEIP$^{-/-}$ lungs (n=6) SP-C protein was decreased while in 40% of KLEIP$^{-/-}$ lungs (n=4) SP-C protein expression was increased. This correlates with lung Hif-2α expression at E18.5 as shown in Fig. 5B. Left: Representative Western blot including its individual quantification. *$p<0.05$.

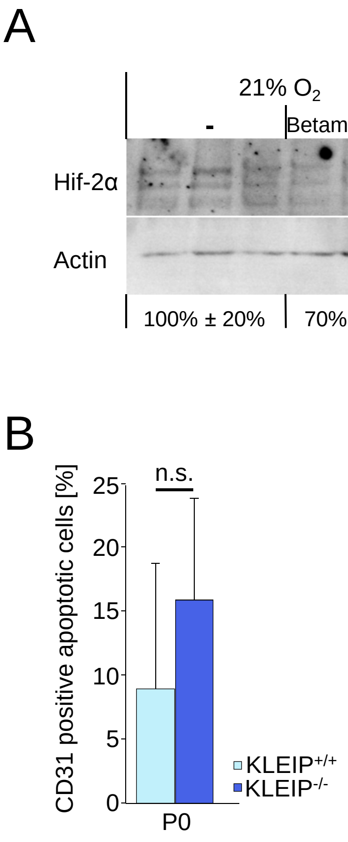

Suppl. Fig. 7: Betamethasone increases endothelial Hif-2α expression and prevents endothelial apoptosis in P0 KLEIP$^{-/-}$ lungs. (A) Betamethasone treatment stimulates Hif-2α expression under hypoxic conditions in cultured endothelial cells. The effect of betamethasone on HUVEC was investigated under normoxic (21%O_2) and hypoxic (0.6%O_2) conditions. While betamethasone did not increase Hif-2α synthesis under normoxic conditions, it strongly enhanced Hif-2α expression under hypoxia; n=3. (B) Quantification of TUNEL/CD31 double positives cells in relation to total TUNEL positive cells after betamethasone treatment in P0 KLEIP$^{-/-}$ lungs did not show significant increased apoptotic rates (n=5 animals per group). n.s.: not significant.
